# Provider Visits for Asthma: Potential Barriers for Insured Children

**DOI:** 10.5539/gjhs.v7n5p96

**Published:** 2015-02-24

**Authors:** Amber M. Goedken, Julie M. Urmie, Linnea A. Polgreen

**Affiliations:** 1 College of Pharmacy, University of Iowa, Iowa City, Iowa, United States

**Keywords:** asthma, barriers, child, national survey, utilization

## Abstract

**Objective:**

The barriers to provider visits for asthma in insured children are not well understood. Our objective was to examine the relationship between parent, family, and child attributes and asthma visits in insured children.

**Methods:**

This retrospective, cross-sectional analysis of 2007 Medical Expenditure Panel Survey-Household Component data included insured children 0-17 years old reported to have active asthma. We summed the number of provider visits during which asthma was treated or diagnosed to represent the frequency of asthma visits during the year. Probit models were used to estimate the relationship between parent, family, and child attributes and asthma visits.

**Results:**

Seventy percent of the 542 children did not have an asthma visit during the year. Children with parents employed full time were 16 percentage points less likely to have an asthma visit than children whose parents were not working (*P* = .01).

**Conclusion:**

Many insured children go more than a year without seeing a provider for their asthma, signaling that insurance is not sufficient to guarantee children will receive asthma monitoring. The attributes related to asthma visits suggest potential barriers that providers might want to consider to increase participation in asthma visits.

## 1. Introduction

Asthma is poorly controlled in approximately half of children with this condition (Hammer et al., 2008; Liu et al., 2010; Schmier et al., 2007). Those with poorly controlled asthma experience limitations in their normal activities, nighttime awakenings, and frequent symptoms requiring use of rescue inhalers (National Heart, 2007). They are more likely to be absent from school than children whose asthma is controlled (Schmier et al., 2007). The risk of exacerbations requiring oral corticosteroids, emergency department visits, and/or hospitalizations is higher in children with poorly controlled asthma than in those with well controlled asthma (Liu et al., 2010; Schmier et al., 2007).

Factors contributing to poorly controlled asthma include suboptimal medication, nonadherence, environmental factors, and comorbidities (Ducharme, 2011). For a provider to identify a patient with uncontrolled asthma, identify the likely cause, and work to resolve that cause, the provider needs to interact with the patient. The National Asthma Education and Prevention Program recommends regular monitoring of children with asthma with a suggested frequency of approximately every six months for those with intermittent or mild persistent asthma who have been well controlled for at least three months and more frequently for those with uncontrolled or severe persistent asthma (National Heart, 2007). This monitoring includes assessments of asthma control, which can lead to interventions to improve inadequate control. Assessments and interventions benefit those with poorly controlled asthma and those with historically well controlled asthma because asthma control can decline over time. Provider monitoring of asthma is particularly important given that patients tend to overestimate their level of control (Hammer et al., 2008). Failure to have a provider visit or extended gaps between visits means children may be unnecessarily suffering from uncontrolled asthma or declining control and increased risk of exacerbation.

Forty-four percent of children with asthma observed for more than a year on average did not visit a provider for the condition (Chen & Escarce, 2008). The importance of asthma visits and the number of children not receiving them necessitates understanding the factors that prevent visits, so they can be addressed. Social cognitive theory proposes that internal and external factors determine an individual’s behavior (Bandura, 2004). The external factors that hinder performance of a behavior are barriers. We are interested in barriers because they are modifiable and can be addressed by providers and policymakers. Lack of health insurance is a known barrier to children’s asthma visits (Stoddard et al., 1994), but it is not the only barrier. Barriers identified in children with asthma include difficulty with appointment times, traveling to visits, and paying for visits (Wood et al., 1993), but we do not know which barriers apply to insured children. This creates a knowledge gap because more than 89% of children with asthma were insured prior to the Affordable Care Act, and the percentage should only increase under the individual insurance mandate (National Center for Environmental Health, 2013). We worked toward closing this gap by assessing whether certain parent, family, and child attributes were related to asthma visits in insured children. We focused on potential barriers to visits, including attributes not previously applied to the study of asthma visits in children: parent health, parent provider visits, parent employment, and parent comfort speaking English.

## 2. Methods

### 2.1 Design, Data Source, and Sample

The study received approval from the university’s institutional review board. The design is a cross-sectional study of families participating in the 2007 Medical Expenditure Panel Survey-Household Component (MEPS-HC), a nationally representative survey of the U.S. civilian noninstitutionalized population that is publicly available for download from the MEPS website. Households in the MEPS-HC are selected from households that participated in the National Health Interview Survey. Households with Hispanic, black, Asian, and low income residents are oversampled. Participating households comprise a panel that is surveyed in five rounds over a two and a half-year period to collect two years of data. The survey is a computer-assisted personal interview survey that is completed by a household member who provides information on all the members of the household. The MEPS-HC data from a given year include data from the second year of one panel and data from the first year of the subsequent panel.

Children ≤17 years old with complete data were included. Subjects were required to have asthma, meaning either (A) a medical provider had diagnosed the child with asthma and the child still had asthma at the end of the year or (B) the child had a medical event, missed school or work, spent the day in bed, or was bothered due to asthma during the year (Kim et al., 2009; Wang et al., 2005). Children had to be insured through the same source of coverage during the entire year. We were unable to measure the amounts families were required to pay for visits and the breadth of provider networks for their plans, so we required children to have the same source of coverage to reduce the number of children that would experience changes in cost-sharing or networks during the year. In the MEPS-HC, children are linked to the biological, step, or adoptive parents living in their households. Only children linked to at least one parent were included. Only one parent was studied. The mother was chosen if she lived in the household during the entire year because mothers are typically responsible for their children’s health care (Wyn et al., 2003). If the mother lived outside the household during the year but the father lived in the household all year, the father was selected.

### 2.2 Utilization Measures

A visit to a medical provider’s office or outpatient department was classified as an asthma visit if asthma was a condition diagnosed or treated at that visit (Wang et al., 2005). The child had to see the provider in person, and urgent visits were excluded. Two binary variables were created after counting the asthma visits. One variable equaled 1 if a child had two or more asthma visits and 0 otherwise (Shields et al., 2004). This measure assessed whether asthma visits reached the minimum suggested quantity for the year and was based on the recommendation that asthma should be monitored every six months (National Heart, 2007). The second variable equaled 1 if a child had one or more asthma visits and 0 otherwise. It captured whether a child had at least one visit with a medical provider for asthma during the year.

### 2.3 Parent, Family, and Child Attributes

We selected attributes that, if related to asthma visits, would suggest potential barriers to those visits for insured children. We included parent health, parent provider visits, parent employment, and parent comfort speaking English, which have not been examined previously for asthma visits but have been studied for children’s provider visits for any type of health care (Davidoff et al., 2003; DeVoe et al., 2009; Hanson, 1998; Newacheck & Halfon, 1986). Also included were parent structure, family income in relation to the Federal Poverty Level, region of residence, residence in a Metropolitan Statistical Area, number of children in the household, child source of coverage, child age, and child health (Chen & Escarce, 2008; Stoddard et al., 1994).

We adjusted for attributes believed to represent the internal factors of knowledge, beliefs, and expectations outlined by social cognitive theory (Bandura, 2004). Again, we included attributes not previously examined for asthma visits but examined for children’s visits for any type of care. These attributes were parent insurance, parent worry about the child’s health relative to other parents, and parent agreement with the statement “I can overcome illness without help from a medically trained person” (Davidoff et al., 2003; DeVoe et al., 2009; Goedken et al., 2014; Hanson, 1998; Janicke et al., 2001). Also included were parent age, parent education, child race/ethnicity, and an indicator for whether the child was the oldest child in the household (Chen & Escarce, 2008; Stoddard et al., 1994).

### 2.4 Data Analysis

Descriptive statistics were produced for the sample. Each of the binary utilization measures were regressed on the measured attributes using probit models. The level of significance for all analyses was α = 0.05. Sample selection and variable recoding were performed using SAS version 9.2. Statistical analyses were performed using Stata/IC version 10.1. Stata survey commands that adjust for the complex sampling design of the MEPS-HC were used for the analyses (Machlin et al., 2005).

## 3. Results

Our sample included 542 children. Weighted to represent the U.S. population, the estimated percentage of children with two or more provider visits for asthma during the year was 14.9%; 70.8% did not have at least one visit (Table 1). Nearly 32% of children were enrolled in Medicaid or the Children’s Health Insurance Program. The majority of children were in good to excellent health and were not limited due to their health. Nearly all the parents we examined were mothers.

**Table 1:** Sample characteristics (*n* = 542)

Variable	Weighted Mean
Parent physical health score (PCS-12)	51.5
Parent mental health score (MCS-12)	49.3
Number of children	2.3
Child age (years)	9.2
Parent age (years)	38.2
	%
Parent use	
No visits	18.2
1-2 visits	24.6
3-6 visits	27.6
>6 visits	29.6
Parent employment	
Not working	32.1
Part time	26.4
Full time	41.5
Parent comfortable speaking English	95.6
Parent structure	
Two parents	68.6
Single mother	28.2
Single father	3.3
Family income	
<100% FPL	20.0
100%-124% FPL	3.4
125%-199% FPL	12.4
200%-399% FPL	30.4
≥400% FPL	33.9
Region of residence	
Northeast	17.8
Midwest	23.0
South	37.7
West	21.5
Lives in Metropolitan Statistical Area	85.4
Child insurance	
Medicaid/Children’s Health Insurance Program	31.9
Private	64.4
Other public	1.1
Multiple sources	2.7
Child health status	
Poor or fair	6.3
Good	27.4
Very good	34.5
Excellent	31.7
Child has limitation due to health	9.4
Parent insured	88.9
Parent worry about child’s health compared to others	
Worries same or less than other parents	58.0
Worries more than other parents	35.0
Don’t know	7.0
Parent self-care expectation	
Strongly disagree can overcome illness w/o medical help	41.6
Neither strongly agree nor strongly disagree	55.7
Strongly agree can overcome illness w/o medical help	2.7
Parent education	
Less than high school	10.4
High school	42.2
Associate degree	16.6
Bachelor’s degree	18.6
Graduate degree	12.2
Child race/ethnicity	
Caucasian	56.5
African American	18.7
Other	8.3
Hispanic	16.5
Oldest child in household	46.4
≥1 visit for asthma	29.2
≥2 visits for asthma	14.9

*Note.* PCS = Physical Component Summary; MCS = Mental Component Summary; FPL = Federal Poverty Level.

The children of single mothers and single fathers were combined into a single category in the multivariable models because none of the 11 children with single fathers had an asthma visit. Compared to children whose parents had earned a bachelor’s degree, children whose parents had not earned a high school or General Educational Development (GED) diploma were 37 percentage points less likely to have an asthma visit, holding all other variables at the mean (Table 2). Children whose parents were employed full time were 16 percentage points less likely to have an asthma visit than children whose parents were not working, and children living in the West were 25 percentage points less likely to have a visit than children in the Northeast. Children in very good or excellent health were less likely to have an asthma visit than children in fair or poor health. Parent education, parent employment, region of residence, and child health status were also related to whether a child had multiple asthma visits (Table 3).

**Table 2:** Coefficients and marginal effects from probit model predicting whether child had ≥1 asthma visit (*n* = 542)

Variable	b	Standard error	p	Marginal effects
Parent physical health score (PCS-12)	-0.01	0.01	0.27	-0.003
Parent mental health score (MCS-12)	0.01	0.01	0.30	0.003
Parent use (no visits)		
1-2 visits	-0.26	0.25	0.30	-0.08
3-6 visits	0.11	0.23	0.63	0.04
>6 visits	-0.15	0.24	0.54	-0.05
Parent employment (not working)		
Part time	-0.03	0.19	0.86	-0.01
Full time	-0.49	0.19	0.01	-0.16
Parent comfortable speaking English	-0.09	0.37	0.80	-0.03
Single parent	-0.32	0.20	0.12	-0.10
Family income (<100% FPL)		
100%-124% FPL	-0.51	0.34	0.13	-0.16
125%-199% FPL	-0.10	0.25	0.69	-0.03
200%-399% FPL	-0.15	0.29	0.60	-0.05
≥400% FPL	-0.19	0.35	0.59	-0.06
Region of residence (Northeast)		
Midwest	-0.32	0.22	0.15	-0.10
South	-0.41	0.20	0.05	-0.13
West	-0.78	0.21	<0.01	-0.25
Lives in Metropolitan Statistical Area	0.03	0.22	0.91	0.01
Number of children	-0.02	0.07	0.80	-0.01
Child insurance (Medicaid/CHIP)		
Private	0.04	0.30	0.89	0.01
Other public	0.46	0.43	0.28	0.15
Multiple sources	-0.46	0.45	0.31	-0.15
Child age (in years)	-0.06	0.02	0.01	-0.02
Child health status (poor or fair)		
Good	-0.37	0.27	0.18	-0.12
Very good	-0.80	0.29	0.01	-0.26
Excellent	-0.73	0.29	0.01	-0.23
Child has limitation due to health	0.45	0.25	0.08	0.14
Parent education (less than high school)		
High school	0.44	0.28	0.11	0.14
Associate degree	0.47	0.32	0.15	0.15
Bachelor’s degree	1.16	0.34	<0.01	0.37
Graduate degree	0.79	0.37	0.03	0.25

*Note.* b = coefficient; p = p-value; PCS = Physical Component Summary; MCS = Mental Component Summary; FPL = Federal Poverty Level; CHIP = Children’s Health Insurance Program. Reference groups are in parentheses. All variables representing potential barriers are included. Also included are statistically significant variables representing internal factors.

**Table 3:** Coefficients and marginal effects from probit model predicting whether child had ≥2 asthma visits (*n* = 542)

Variable	b	Standard error	p	Marginal effects
Parent physical health score (PCS-12)	-0.002	0.01	0.82	-0.0004
Parent mental health score (MCS-12)	0.003	0.01	0.74	0.001
Parent use (no visits)		
1-2 visits	-0.13	0.28	0.64	-0.02
3-6 visits	-0.02	0.28	0.95	-0.003
>6 visits	0.20	0.29	0.49	0.04
Parent employment (not working)		
Part time	-0.19	0.22	0.37	-0.03
Full time	-0.72	0.22	<0.01	-0.13
Parent comfortable speaking English	-0.04	0.39	0.92	-0.01
Single parent	0.22	0.23	0.34	0.04
Family income (<100% FPL)		
100%-124% FPL	-0.38	0.41	0.35	-0.07
125%-199% FPL	0.11	0.28	0.70	0.02
200%-399% FPL	-0.05	0.30	0.88	-0.01
≥400% FPL	-0.16	0.39	0.69	-0.03
Region of residence (Northeast)		
Midwest	-0.75	0.26	0.01	-0.13
South	-0.80	0.24	<0.01	-0.14
West	-1.08	0.24	<0.01	-0.19
Lives in Metropolitan Statistical Area	-0.01	0.25	0.97	-0.002
Number of children	-0.07	0.08	0.35	-0.01
Child insurance (Medicaid/CHIP)		
Private	0.42	0.34	0.21	0.07
Other public	1.48	0.47	<0.01	0.26
Multiple sources	-0.26	0.48	0.59	-0.05
Child age (in years)	-0.04	0.03	0.14	-0.01
Child health status (poor or fair)		
Good	-0.94	0.33	0.01	-0.17
Very good	-1.04	0.33	<0.01	-0.19
Excellent	-1.20	0.34	<0.01	-0.21
Child has limitation due to health	0.53	0.26	0.05	0.09
Parent education (less than high school)		
High school	0.45	0.25	0.08	0.08
Associate degree	0.69	0.36	0.06	0.12
Bachelor’s degree	0.96	0.41	0.02	0.17
Graduate degree	0.80	0.41	0.05	0.14

*Note.* b = coefficient; p = p-value; PCS = Physical Component Summary; MCS = Mental Component Summary; FPL = Federal Poverty Level; CHIP = Children’s Health Insurance Program. Reference groups are in parentheses. All variables representing potential barriers are included. Also included are statistically significant variables representing internal factors.

## 4. Discussion

Strict interpretation of the National Asthma Education and Prevention Program guidelines indicates at least two visits per year are recommended for any child with asthma (National Heart, 2007). A looser interpretation says at least two visits are recommended for children with poorly controlled asthma but fewer visits may be appropriate for children with well controlled asthma; the guidelines acknowledge the importance of the clinician’s judgment in setting the exact schedule of visits. Given that approximately half of children with asthma have poorly controlled asthma, we expected ≥50% of children in our study to have at least two asthma visits (Hammer et al., 2008; Liu et al., 2010; Schmier et al., 2007). However, only 15 percent had two or more visits, and only 30 percent had at least one. Another study found that from 1996 to 2000, less than 60% of children had a visit for asthma in a two-year period (Kim et al., 2009). Our results indicate a persistent lack of asthma visits and recommended asthma monitoring, even among insured children.

The attributes related to asthma visits in insured children suggest potential barriers that are not alleviated by insurance. One such barrier is inconvenient appointment times. Appointment times that conflict with parental responsibilities make attendance difficult. This may be particularly true for employed parents. Parents employed full time may have difficulty finding available visits outside of working hours or leaving work to attend visits. We found children of parents employed full time were less likely to receive asthma visits, a relationship which has not been examined previously even in the general pediatric population with asthma. Parents of mostly uninsured children with asthma have identified difficulty with appointment times as a barrier to care (Wood et al., 1993), and our results suggest this may be a barrier for insured children as well. Further demonstrating that employed parents may struggle to juggle other responsibilities with caring for children with asthma, maternal employment is associated with poorer asthma control (Bloomberg et al., 2009) and greater likelihood of an asthma episode (Morrill, 2011). Providers may want to consider offering more convenient appointments for employed parents, such as during evenings and/or weekends to accommodate work schedules.

Children living in the Northeast are more likely to see a provider for asthma than children living in the West. Other studies have noted higher rates of utilization among children in the Northeast compared to other regions (De Voe et al., 2009; Goedken et al., 2014). This may reflect greater access due to a higher concentration of pediatricians in the Northeast (Chang & Halfon, 1997). Even with insurance, parents must be able to find providers who can care for their children, which should be easier with more providers in an area. The potential barrier suggested by these results is a lack of available providers, an issue for consideration by policymakers.

Another potential barrier is limited contact with health care providers. Child health status was related to asthma visits, whereby children in better health were less likely to have asthma visits than children in poorer health. Children in better health are expected to have fewer visits to providers for other illnesses than their counterparts (Newacheck & Halfon, 1986). Fewer contacts with providers mean fewer opportunities for asthma to be addressed. The decreased likelihood of asthma visits among children in better health signals fewer contacts as a potential barrier to asthma visits. Providers may need to consider implementing or increasing outreach to their healthier patients.

In addition to suggesting a potential barrier, the relationship between health status and visits suggests health status may be an indicator of the child’s asthma control. Based on guideline recommendations (National Heart, 2007), we expect a greater likelihood of asthma visits in children with more severe or poorly controlled asthma than in children with less severe or well controlled asthma, but we were unable to measure asthma severity or control. Poorly controlled asthma is expected to be concentrated among children in poorer health because a child whose asthma is making normal activities difficult is unlikely to be reported as having excellent health. The increased likelihood of visits among those in poorer health compared to those in better health supports the suggestion that health status reflects asthma control.

Our results also suggest a possible internal factor driving asthma visits in insured children. Incorrect understanding of asthma could hinder parents from pursuing asthma monitoring visits for their children. For example, some parents erroneously perceive asthma as an acute rather than chronic illness (Bokhour et al., 2008). Poor asthma knowledge is more concentrated among less educated parents (Radic et al., 2014; Zhao et al., 2002). We found that visits were less likely among children whose parents did not earn a high school diploma or its equivalent compared to children whose parents earned a college degree. The decreased likelihood of visits in families with presumably poorer knowledge of asthma supports the proposition that parent knowledge may play a role in asthma visits for insured children. Providers spend time educating parents about asthma but even greater efforts to address knowledge deficits and inaccuracies may be needed.

One of the limitations of using the MEPS-HC as our data source is our inability to determine how many asthma visits were recommended by children’s providers and the recommended schedule for those visits. Because of this, we could not evaluate whether children were not meeting their providers’ recommendations. We could only assess their receipt of a minimum number of visits. For example, a child’s provider may have wanted to see the child every three months, but the child may have only presented for two of the four recommended visits. Even though that child was not getting the care the provider wanted, that child would have met the minimum quantities in our analyses. Another limitation of the MEPS-HC is data are collected retrospectively and are based on self-report. The reasons for visits are not verified with medical records or administrative claims. Some self-reported MEPS-HC data are supplemented with data collected from respondents’ medical providers, but this information is not. Parents could make errors in reporting. If a provider assessed a child’s asthma at a visit without the parent being aware of it, the parent would not report that visit as being for asthma. Parents could fail to mention asthma as a reason for a visit, or they could forget to mention a visit altogether. The design of the MEPS-HC makes forgetting less likely as respondents are asked to report events over the last several months rather than over the last year. It is easier for respondents to remember events accurately if they occurred recently (Macek et al., 2002).

Despite having insurance, a substantial portion of children with asthma do not visit a provider for this condition during the year. These results were similar to previous research among insured and uninsured children (Kim et al., 2009). The failure of children to receive asthma visits is concerning because it does not give providers the opportunity to monitor children’s asthma, which could contribute to uncontrolled asthma and preventable asthma exacerbations. To address this problem, providers and policymakers need to understand the barriers to children’s provider visits for asthma. This study investigated whether certain parent, family, and child attributes were related to asthma visits in insured children. Children of parents employed full time, children in very good or excellent general health, and those living outside of the Northeast region of the U.S. were less likely to have one or two provider visits during the year. These attributes suggest potential barriers faced by insured children. Recognition of these potential barriers is the first step toward increasing participation in asthma visits. Future studies could interview or survey the parents of insured children to confirm the exact barriers these families encounter and determine what would make it easier for them to attend asthma visits.

## References

[ref1] Bandura A (2004). Health promotion by social cognitive means. Health Education and Behavior.

[ref2] Bloomberg G. R, Banister C, Sterkel R, Epstein J, Bruns J, Swerczek L, Garbutt J. M (2009). Socioeconomic, family, and pediatric practice factors that affect level of asthma control. Pediatrics.

[ref3] Bokhour B. G, Cohn E. S, Cortes D. E, Yinusa-Nyahkoon L. S, Hook J. M, Smith L. A, Lieu T. A (2008). Patterns of concordance and non-concordance with clinician recommendations and parents’ explanatory models in children with asthma. Patient Education and Counseling.

[ref4] Chang R. K, Halfon N (1997). Geographic distribution of pediatricians in the United States: An analysis of the fifty states and Washington, DC. Pediatrics.

[ref5] Chen A. Y, Escarce J. J (2008). Family structure and the treatment of childhood asthma. Medical Care.

[ref6] Davidoff A, Dubay L, Kenney G, Yemane A (2003). The effect of parents’ insurance coverage on access to care for low-income children. Inquiry.

[ref7] DeVoe J. E, Tillotson C. J, Wallace L. S (2009). Children’s receipt of health care services and family health insurance patterns. Annals of Family Medicine.

[ref8] Ducharme F. M (2011). Leukotriene receptor antagonists as first line or add-on treatment for asthma. British Medical Journal.

[ref9] Goedken A. M, Urmie J. M, Polgreen L. A (2014). Factors related to receipt of well-child visits in insured children. Maternal and Child Health Journal.

[ref10] Hammer S. C, Robroeks C. M, van Rij C, Heynens J, Droog R, Jobsis Q, Dompeling E (2008). Actual asthma control in a paediatric outpatient clinic population: Do patients perceive their actual level of control?. Pediatric Allergy and Immunology.

[ref11] Hanson K. L (1998). Is insurance for children enough? The link between parents’ and children’s health care use revisited. Inquiry.

[ref12] Janicke D. M, Finney J. W, Riley A. W (2001). Children’s health care use: A prospective investigation of factors related to care-seeking. Medical Care.

[ref13] Kim H, Kieckhefer G. M, Greek A. A, Joesch J. M, Baydar N (2009). Health care utilization by children with asthma. Preventing Chronic Disease.

[ref14] Liu A. H, Gilsenan A. W, Stanford R. H, Lincourt W, Ziemiecki R, Ortega H (2010). Status of asthma control in pediatric primary care: Results from the pediatric Asthma Control Characteristics and Prevalence Survey Study (ACCESS). Journal of Pediatrics.

[ref15] Macek M. D, Manski R. J, Vargas C. M, Moeller J. F (2002). Comparing oral health care utilization estimates in the United States across three nationally representative surveys. Health Services Research.

[ref16] Machlin S, Yu W, Zodet M (2005). Computing standard errors for MEPS estimates.

[ref17] Morrill M. S (2011). The effects of maternal employment on the health of school-age children. Journal of Health Economics.

[ref18] National Center for Environmental Health (2013). Insurance coverage and barriers to care for people with asthma.

[ref19] National Heart, Lung, and Blood Institute (2007). Expert Panel Report 3: Guidelines for the diagnosis and management of asthma (EPR-3).

[ref20] Newacheck P. W, Halfon N (1986). The association between mother’s and children’s use of physician services. Medical Care.

[ref21] Radic S. D, Milenkovic B. A, Gvozdenovic B. S, Zivkovic Z. M, Pesic I. M, Babic D. D (2014). The correlation between parental education and their knowledge of asthma. Allergologia et Immunopathologia.

[ref22] Schmier J. K, Manjunath R, Halpern M. T, Jones M. L, Thompson K, Diette G. B (2007). The impact of inadequately controlled asthma in urban children on quality of life and productivity. Annals of Allergy, Asthma & Immunology.

[ref23] Shields A. E, Comstock C, Weiss K. B (2004). Variations in asthma care by race/ethnicity among children enrolled in a state Medicaid program. Pediatrics.

[ref24] Stoddard J. J, St. Peter R. F, Newacheck P. W (1994). Health insurance status and ambulatory care for children. New England Journal of Medicine.

[ref25] Wang L. Y, Zhong Y, Wheeler L (2005). Direct and indirect costs of asthma in school-age children. Preventing Chronic Disease.

[ref26] Wood P. R, Hidalgo H. A, Prihoda T. J, Kromer M. E (1993). Hispanic children with asthma: Morbidity. Pediatrics.

[ref27] Wyn R, Ojeda V, Ranji U, Salganicoff A (2003). Women, work, and family health: A balancing act.

[ref28] Zhao X, Furber S, Bauman A (2002). Asthma knowledge and medication compliance among parents of asthmatic children in Nanjing, China. Journal of Asthma.

